# Vapor of Ether in Asthma and Hooping Cough

**Published:** 1847-06

**Authors:** 


*Vapor of Ether in Asthma and Hoopingcough.*—Dr. Willis makes the following observations on the subject in the *Medical Gazette:*

"Ether, given by the mouth, has long been familiarly employed in the treatment of asthma. I have for many years been aware of the fact that it is vastly more efficacious administered directly in vapor by the breath. My plan of using it is extremely simple. I have had recourse to no kind of apparatus for this purpose, but have been content to pour two, three, or four drachms of the fluid upon a clean handkerchief, and to direct this to be held closely to the mouth and nostrils; a single short and difficult inspiration is hardly made before the effect is experienced; and I have occasionally seen the paroxysm ended in six or eight minutes, the respiration having in that brief interval become almost natural.

"It is not otherwise with hooping-cough; the paroxysms of coughing are positively cut short by having the ether and the handkerchief in readiness, and using them when the fit is perceived to be coming on. So effectual have I seen their immediate application, that I have even found it necessary to suffer the patient to have an occasional fit of coughing to its natural termination, with a view of clearing the chest from accumulated mucus."—*Ib.*

